# Ioning out glioblastoma: ferroptosis mechanisms and therapeutic frontiers

**DOI:** 10.1038/s41420-025-02711-6

**Published:** 2025-08-26

**Authors:** Hetong Sun, Jiayu Zhang, Henan Qi, Dandan Jiang, Caofang Hu, Chengyu Mao, Wei Liu, Hongzhao Qi, Jinbao Zong

**Affiliations:** 1 Clinical Laboratory and Qingdao Key Laboratory of Immunodiagnosis, Qingdao Hiser Hospital Affiliated of Qingdao University (Qingdao Traditional Chinese Medicine Hospital), Qingdao, China; 2 Institute for Translational Medicine, The Affiliated Hospital of Qingdao University, College of Medicine, Qingdao University, Qingdao, China; 3 Department of Pulmonary and Critical Care Medicine, Qilu Hospital of Shandong University, Jinan, Shandong China; 4 Breast Disease Center, The Affiliated Hospital of Qingdao University, Qingdao, China; 5 Department of Emergency Medicine, The Affiliated Hospital of Qingdao University, College of Medicine, Qingdao University, Qingdao, China

**Keywords:** Targeted therapies, CNS cancer

## Abstract

Glioblastoma (GBM) (IDH-wildtype), the most prevalent and malignant primary brain tumor in adults, continues to pose a major therapeutic challenge in neuro-oncology. Despite significant advancements in cancer diagnosis and treatment technologies, conventional therapies remain largely ineffective against this tumor, urgently necessitating breakthrough treatment strategies. This comprehensive review critically examines recent advances in targeting ferroptosis, an iron-dependent form of non-apoptotic cell death mediated through reactive oxygen species (ROS) accumulation and lipid membrane peroxidation, for therapeutic intervention in GBM. The key aspects analyzed encompass the unique molecular mechanisms that distinguish ferroptosis from apoptosis and necrosis, along with its regulatory networks in GBM. The analysis also explores the therapeutic potential of targeting critical ferroptosis pathways, including dysregulated iron metabolism, impaired antioxidant defenses, and abnormal lipid peroxidation. Additionally, it examines the synergistic effects and molecular basis of combining ferroptosis inducers with chemo-radiotherapy or immunotherapy. Finally, the study highlights innovative applications of nano-drug delivery technologies in overcoming blood-brain barrier (BBB) limitations and enhancing the precision of ferroptosis-targeted therapy. Notably, this review provides a comprehensive analysis of the interplay between ferroptosis regulation and the tumor immune microenvironment, highlighting a promising ‘ferroptosis-immunotherapy’ combination strategy with clinical translation potential for GBM treatment. While challenges persist regarding incomplete understanding of regulatory networks and nanocarrier biosafety issues, this review not only provides a theoretical framework for comprehending ferroptosis-mediated anti-GBM mechanisms but also outlines future research directions, including in-depth dissection of ferroptosis signaling hubs, development of intelligent nano-delivery systems, and establishment of preclinical safety evaluation protocols. These findings are expected to provide revolutionary therapeutic targets for achieving precision treatment of GBM.

## Facts

- Inducing ferroptosis effectively eliminates tumor cells and can also trigger immunogenic cell death (ICD). However, excessive ferroptosis may result in hypoxia, nutrient deprivation, and inflammation-related immunosuppression within the tumor microenvironment (TME), potentially facilitating tumor growth. The specific threshold and mechanisms that govern the transition from therapeutic to detrimental effects remain unclear. Future research must delineate this threshold and devise strategies, such as controlled dosing, microenvironment modulation, and combination therapies, to remain within the therapeutic window.
- Resistance mechanisms, such as GPX4 upregulation, compensatory FSP1 activity, and the protective role of specific TME components (e.g., M2 macrophages and Tregs via GPX4 expression), limit the efficacy of ferroptosis. The extreme heterogeneity of glioblastoma multiforme (GBM), including genetic, epigenetic, and metabolic diversity, further complicates therapeutic targeting. Urgent research is required to identify synergistic combinations (e.g., FSP1 inhibitors with GPX4 inhibitors, ferroptosis inducers with IDO1/m6A inhibitors, and ferroptosis inducers with immune checkpoint blockade) that can simultaneously bypass multiple resistance pathways across diverse GBM subclones.
- While nanomedicine offers promising solutions for blood-brain barrier (BBB) penetration and targeted delivery, significant challenges remain. These include potential immunogenicity, undefined long-term biosafety profiles of novel nanomaterials, suboptimal drug loading/release kinetics, limited penetration into the invasive tumor margin, and overcoming the immunosuppressive/hypoxic/acidic TME. Research must focus on intelligent, stimuli-responsive, biomimetic, and potentially multifunctional (theranostic) nanoplatforms with rigorous safety evaluation.

## Introduction

Glioma is the most prevalent primary brain tumor, originating from the malignant transformation of glial cells in the brain and spinal cord [1, 2]. According to WHO CNS5 (2021), glioblastoma (GBM), typically represents the most aggressive form of glioma, is primarily defined as an IDH-wildtype diffuse astrocytic glioma with CNS WHO grade four histological or molecular features [3]. Epidemiological data indicate that GBM accounts for over 50% of malignant CNS tumors in adults [4]. A report by the Global Institute of Oncology of the World Health Organization revealed that in 2020, in China, there were nearly 79,000 new cases of primary intracranial tumors and 65,000 deaths. Standard GBM treatment involves maximal safe surgical resection followed by concurrent radiotherapy and chemotherapy [5, 6]. However, GBM’s frequent location in eloquent brain regions (e.g., language, motor, sensory) and its infiltrative nature limit radical resection. Residual tumor cells often persist, leading to progression or recurrence. Furthermore, radioresistance and damage to normal brain tissue from radiotherapy, coupled with chemotherapy limitations including poor blood-brain barrier (BBB) penetration, drug resistance, and significant side effects, collectively reduce overall treatment efficacy [7]. Consequently, there is currently no effective clinical treatment for GBM, resulting in a 5-year survival rate of less than 5% [2]. Investigating novel therapeutic strategies for GBM is both imperative and urgently required to address the limitations of current treatment modalities [8].

Ferroptosis is a regulated cell death (RCD) mechanism characterized by iron-dependent lethal lipid peroxide accumulation [9]. Unlike autophagy or apoptosis, ferroptosis is specifically triggered by dysregulated lipid metabolism and impaired antioxidant defense, not by activation of established pathways like caspase. GBM cells exhibit dysregulated iron metabolism (transferrin receptor overexpression, impaired iron efflux) and altered lipid metabolism (changes in desaturase activity, membrane phospholipid remodeling), conferring heightened ferroptosis susceptibility compared to normal neural cells [10]. Consequently, ferroptosis represents a potential therapeutic target for GBM. In vitro and preclinical studies demonstrate that inhibiting key regulators (e.g., GPX4) effectively induces ferroptosis, reducing GBM cell viability and tumor growth [11]. Ferroptosis-inducing combination therapies (e.g., inducers like erastin or RSL3 with temozolomide) also show promise in enhancing conventional treatments (chemotherapy, radiotherapy) and overcoming resistance [12].

Therefore, the ferroptosis-GBM association offers opportunities to develop novel therapeutic strategies. Elucidating its precise mechanisms in GBM may enable more effective, less toxic treatments for this lethal disease. However, challenges remain, including fully defining ferroptosis regulation and addressing potential off-target effects of inducers. Future research must overcome these limitations to realize the potential of ferroptosis-based GBM therapies.

This review summarizes GBM pathogenesis and ferroptosis mechanisms, then focuses on ferroptosis-targeted GBM therapy through target sites and pharmacological agents. It aims to highlight the therapeutic promise of ferroptosis and stimulate research toward novel ferroptosis-based strategies.

## The pathogenesis of GBM

The pathogenesis of GBM includes gene mutations, signaling pathway dysregulation, epigenetic modifications, and tumor microenvironment (TME), which collectively drive tumor initiation, progression, invasion, and treatment resistance [13, 14]. Genetic mutations play a central role in the pathogenesis of GBM [15]. The most common genetic alterations in GBM involve the inactivation of tumor suppressor genes, such as p53, PTEN, and retinoblastoma (RB1), leading to uncontrolled cell proliferation, escape from apoptosis, and genomic instability [14, 16].

Sun et al. demonstrated that p53 protein suppresses tumorigenesis by inhibiting uncontrolled cell proliferation. BRD8 binds histone H2AZ via a key-lock mechanism, repressing p53 activation and promoting GBM progression; targeting BRD8 reactivates p53 and inhibits tumors [17]. PTEN regulates glycolytic metabolism via the PI3K-AKT pathway to suppress tumors. Xu et al. revealed that PTEN-mediated PGK1 dephosphorylation inhibits glycolysis, ATP production, and proliferation [18]. In GBM, PTEN loss elevates PGK1 phosphorylation, correlating with poor prognosis [19]. AndTP53 mutations, EGFR amplification, and replication stress-induced DNA methylation collectively downregulate RB1 expression [20, 21].

In addition to genetic mutations, dysregulation of signaling pathways is a significant characteristic of GBM pathogenesis. Key pathways such as PI3K/AKT/mTOR (PAM), RAS/RAF/MEK/ERK [22], and Notch are frequently activated in GBM, promoting cell proliferation, survival, and invasion, while also contributing to the resistance of GBM cells to chemotherapy and radiation therapy. The PAM pathway centrally regulates proliferation, epithelial-mesenchymal transition, metabolism, and angiogenesis. Disrupting key components of PAM/Wnt/β-catenin signaling may impair tumor cell function and improve survival [23]. Hyperactive Notch signaling in GBM maintains stemness and confers therapy resistance by blocking differentiation [24–28]. Bazzoni et al. established its role in regulating neural/glioma stem cells (GSCs) [29]. Notch component overexpression associates with high-grade glioma and poor prognosis [30]. HAN et al. linked NOTCH1 overexpression to reduced overall survival in GBM patients [31]; NOTCH1/HES1 upregulation in GSCs drives invasion and recurrence [32].

Furthermore, the TME critically influences GBM pathogenesis via crosstalk among immune cells, stromal cells, and ECM components [33]. Immune cells, such as tumor-associated macrophages (TAMs) and regulatory T cells (Tregs), promote immunosuppression and tumor growth. Tregs mediate immunosuppression by expressing FOXP3 to downregulate NFAT/NF-κB signaling, thereby inhibiting effector cytokines (e.g., IL-2) and immune responses [34]. Secretion of immunosuppressive cytokines (e.g., IL-10, TGF-β) further potentiates suppression [35]. Tregs also highly express immune checkpoint molecules (e.g., CTLA-4, PD-1, GITR), which bind receptors on immune cells to inhibit effector activity [36]. Altered ECM composition (e.g., elevated HA, fibronectin) increases stiffness, facilitating invasion and angiogenesis [37, 38]. Enhanced migration is driven by CD44 overexpression and ECM accumulation [39]. MSCs secrete IL-6, CXCL1/2, and MMPs to degrade ECM and promote metastasis [40, 41]. Increased ECM stiffness engages innate immunity (e.g., tenascin C-CD47 phagocytic signaling in macrophages), underscoring immune regulation in the TME [42].

Tumor heterogeneity in GBM arises from genetic/epigenetic diversity. Epigenetic mechanisms (DNA methylation, histone modifications) dynamically regulate gene expression to foster heterogeneity [43]. Cellular origins determine phenotypic variation, with sub-epigenomes shaping diversity [44]. Intratumoral genetic heterogeneity drives therapeutic resistance and recurrence. Age is a key risk factor: incidence rises after age 54, peaking at 75–84 years (15–24 cases/100,000 population), with a median diagnosis age of 64 [45]. Immune mechanisms contribute to pathogenesis (e.g., radiotherapy-induced resistance via immunomodulation) [46, 47]. Neurocarcinogens (e.g., chemicals, viruses) also promote GBM. Human cytomegalovirus (HCMV) detected in resected tissues implicates its role in tumor activation [48].

**Fig. 1:**
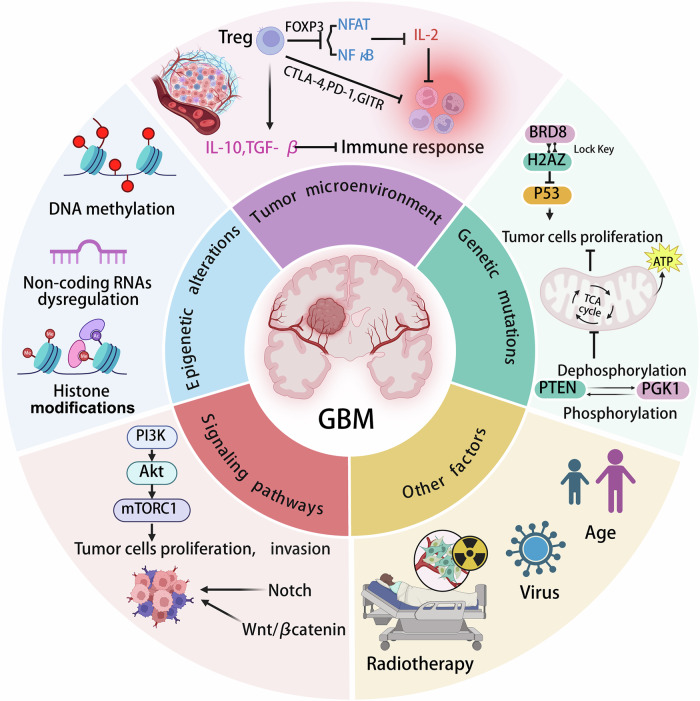
Pathogenic mechanisms of GBM: the pathogenesis of GBM includes genetic mutations, signaling pathway dysregulation, epigenetic alterations, and TME interactions. Genetic mutations include genetic mutations in key regulatory genes, such as P53 and PTEN, but also epigenetic modifications, such as DNA methylation, histone modifications, and non-coding RNA dysregulation. Dysregulation of some signaling pathways, such as Notch, Wnt/β-catenin, and PI3K-AKT, can lead to tumor proliferation and spread. TME is driven by FoxP3-expressing Tregs and immune checkpoint PD-1/CTLA-4, as well as cytokine secretion of IL-10 and TGF-β to suppress antitumor immunity. External factors include aging, radiation therapy, and viral infections like HCMV, which together promote tumor progression and treatment resistance.

In summary, we comprehensively summarize current knowledge on GBM pathogenesis, including genetic mutations, epigenetic alterations, dysregulated signaling pathways, TME, and other contributing factors (Fig. 1). Despite significant advances in elucidating GBM pathogenesis, further research is required to deepen understanding of its underlying mechanisms. Notably, additional studies should address tumor heterogeneity, complex interactions within the TME, and mechanisms of therapeutic resistance.

## Ferroptosis and its key targets in GBM

**Fig. 2:**
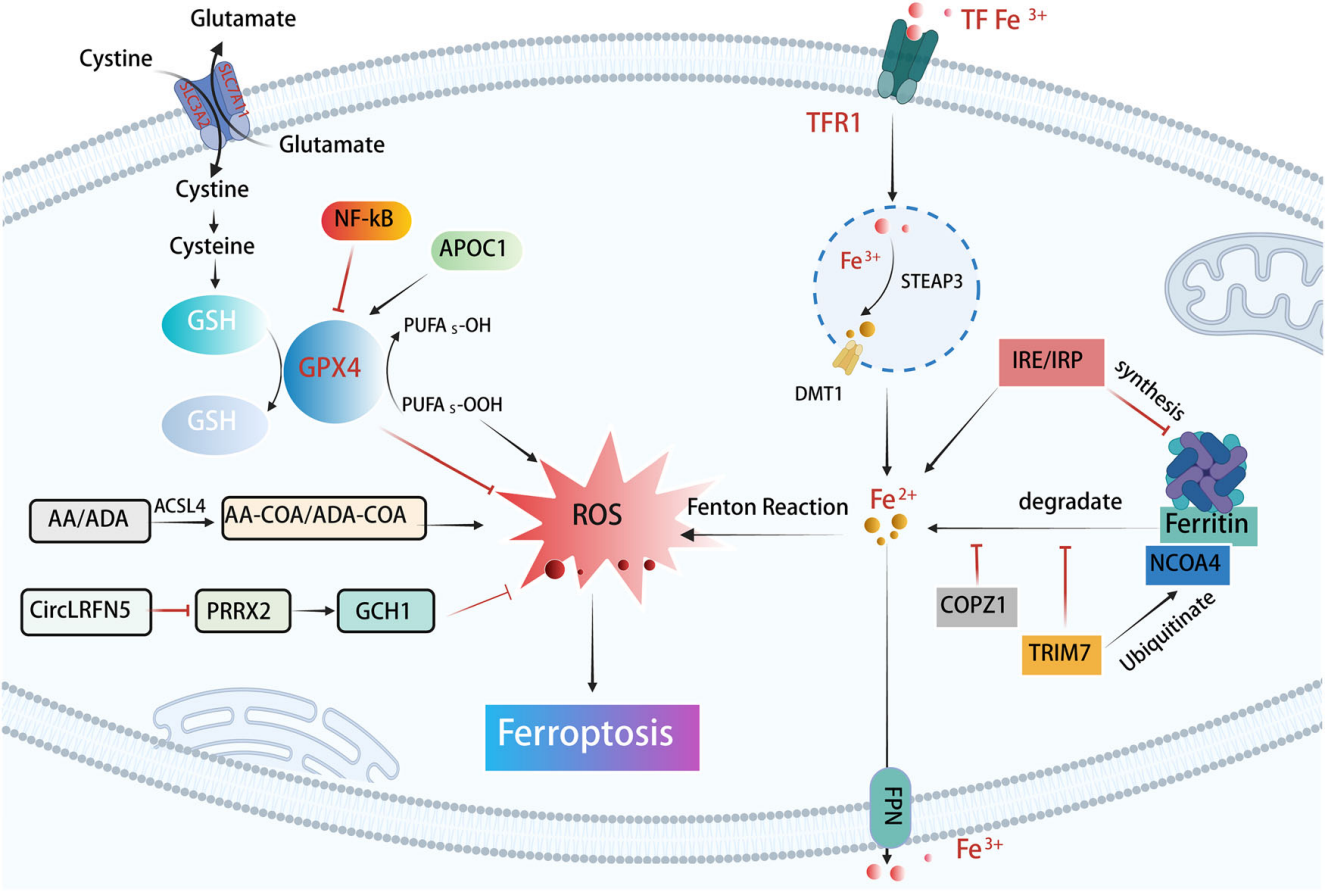
Key ferroptosis targets in GBM: ferroptosis is closely linked to lipid ROS production, which is regulated by both GPX4 levels and iron metabolism. In iron regulation, TRIM7 and COPZ1 suppress iron-related protein expression or activity, reducing cellular iron levels, whereas the IRP/IRE pathway inhibits ferritin degradation, leading to iron accumulation. In GPX4-related regulation, APOC1 enhances GSH levels to promote GPX4 synthesis, while the NF-κB pathway synergistically amplifies ferroptosis under GPX4-deficient conditions. Furthermore, ACSL4 drives AA-CoA/ADA-CoA generation, and circLRFN5 elevates lipid ROS by suppressing the PRRX2/GCH1/BH4 axis. Together, these mechanisms collectively promote ferroptosis in GBM.

Ferroptosis is a unique form of RCD characterized by iron-dependent lipid peroxidation and oxidative stress, distinct from apoptosis or necrosis in terms of morphology, biochemistry, and genetic features. This process is driven by the accumulation of ROS and the peroxidation of polyunsaturated fatty acids in cellular membranes, leading to the formation of lipid hydroperoxides that compromise membrane integrity and cause cell swelling and death. Numerous studies have demonstrated that inducing ferroptosis in GBM cells can effectively inhibit tumor growth and enhance the efficacy of conventional therapies such as chemotherapy and radiation. This section will highlight several key ferroptosis targets, as illustrated in Fig. 2.

GSH, a critical antioxidant, neutralizes ROS and protects cells from oxidative stress. Cysteine, essential for GSH biosynthesis, and its oxidized form, cystine, are integral to this process, and impaired cysteine uptake or defective conversion to cysteine increases ferroptosis vulnerability [49]. System Xc-, composed of SLC7A11 and SLC3A2, imports cysteine for GSH synthesis, and its inhibition reduces GSH levels, leading to ferroptosis. SLC7A11 is essential for cellular resistance to ferroptosis, and its inhibition represents a potential therapeutic target. Research has shown that SLC7A11 promotes the transformation and carcinogenicity of oncogenic KRAS while also reducing oxidative stress in the TME [50]. Elevated extracellular glutamate impairs System Xc- function, depleting GSH and inducing ferroptosis. The tumor suppressor p53 also regulates ferroptosis by modulating iron metabolism and antioxidant pathways. p53 influences iron-related genes like TfR and ferritin, and its activation can reduce TfR1 levels, promoting ferroptosis. p53 also interacts with GPX4 and NRF2, altering cellular sensitivity to ferroptosis inducers. Additionally, p53 inhibits SLC7A11, reducing GSH synthesis and suppressing GPX4 activity. However, the relationship between p53 and ferroptosis is context-dependent, with p53 sometimes exerting protective effects. For example, wild-type p53 colorectal cancer cells resist ferroptosis, while p53 mutations restore sensitivity, as shown by Xie et al. [51]. This dynamic interplay underscores the complexity of ferroptosis regulation and its potential therapeutic applications in diseases like cancer and neurodegeneration. Therefore, exerting the role of the antioxidant defense system can also help promote the ferroptosis process of GBM. Apolipoprotein C1 (APOC1) confers ferroptosis resistance by activating NRF2 and enhancing GSH synthesis through cystathionine β-synthase (CBS) upregulation [52]. In GSCs, the PRRX2/GCH1/BH4 axis provides additional protection against ferroptosis. PRRX2 promotes GSC maintenance [53] while GCH1-derived tetrahydrobiopterin (BH4) exerts antioxidant effects through lipid remodeling [54, 55]. The circular RNA circLRFN5 counteracts this pathway by promoting PRRX2 degradation, whereas SPY1 protein enhances GCH1 translation to bolster BH4 production. In addition, Matesanz-Sánchez et al.‘s study showed that a decrease in EXT2 levels can cause dysregulation of lipid metabolism and antioxidant capacity by altering SAM and transsulfuration pathway enzymes levels, triggering lipid peroxidation and ultimately leading to ferroptosis in GBM cells [12]. Some regulatory pathways can act simultaneously through multiple targets. For example, the Nrf2-ARE pathway serves as a major ferroptosis resistance mechanism. Under oxidative stress, NRF2 escapes KEAP1-mediated degradation [56], translocates to the nucleus, and activates antioxidant genes including HO-1 and NQO1. This pathway maintains redox homeostasis by regulating GSH synthesis and iron metabolism, thereby protecting GBM cells from ferroptosis death. Iron homeostasis is central to ferroptosis regulation, with disruptions significantly influencing cell death. Iron chelators like deferoxamine (DFOM) reduce cellular iron levels and inhibit ferroptosis, as demonstrated by Yang et al. [57]. Ferritin, an iron storage protein, maintains iron balance by binding excess iron to prevent ROS generation or releasing stored iron during deficiency. Autophagy-mediated ferritin degradation, facilitated by the cargo receptor NCOA4, enhances ferroptosis, while decreased NCOA4 expression lowers cellular GSH levels and increases susceptibility [58]. Transferrin (Tf) mediates iron delivery to cells via the transferrin receptor (TfR), and disruption of this process can lead to iron overload and ferroptosis. Regulatory factors like heme oxygenase 1 (HO-1) and Poly(rC) binding proteins (PCBPs) also influence iron homeostasis, with HO-1 overexpression reducing ROS and lipid peroxidation to inhibit ferroptosis, as shown by Villalpando-Rodriguez et al. [59]. Jiang et al. demonstrated that iron chaperones PCBP1/2 mediate iron dysregulation, linking GSH depletion to ferroptosis [60]. Therefore, changes in iron homeostasis can be used as a ferroptosis target for GBM. Li et al. demonstrated that the tripartite motif-containing protein 7 (TRIM7), an E3 ubiquitin ligase, promotes GBM progression by inhibiting ferroptosis through NCOA4 ubiquitination, which reduces ferritinophagy and intracellular free iron levels [61]. Conversely, TRIM7 silencing elevates ROS and iron concentrations, triggering iron-dependent cell death. Similarly, coatomer protein complex subunit zeta 1 (COPZ1) is overexpressed in GBM [32], and its inhibition induces ferroptosis via NCOA4-mediated ferritin degradation, leading to Fe²⁺ accumulation and ROS production through the Fenton reaction. Studies by Zhang et al. have demonstrated that COPZ1 knockdown elevates ROS levels, Fe^2+^ concentrations, and lipid peroxidation [62]. Furthermore, COPZ1 knockdown promotes ferritinophagy and the release of free iron by downregulating ferritin heavy chain 1 (FTH1) expression and upregulating NCOA4 expression, thereby triggering ferroptosis [63]. The COPZ1/NCOA4/FTH1 axis thus presents a promising therapeutic target for GBM treatment. Iron homeostasis is further modulated by the IRP-IRE signaling pathway, where iron regulatory proteins (IRP1/2) bind to iron-responsive elements (IREs) in the untranslated regions of ferritin and ferroportin (FPN) mRNAs. Under iron-deficient conditions, IRPs suppress the translation of these proteins, increasing labile iron pools and sensitizing cells to ferroptosis through enhanced lipid peroxidation [64].

GPX4, a selenoprotein enzyme, is vital for protecting cells against oxidative stress by reducing lipid hydroperoxides and inhibiting lipid peroxidation. As a key defense against ferroptosis, GPX4 prevents lipid peroxide accumulation and maintains membrane integrity. GPX4 deficiency increases ferroptosis susceptibility, as seen in systemic lupus erythematosus, where suppressed GPX4 induces neutrophil ferroptosis [65]. Conversely, Kinowaki et al. demonstrated that GPX4 overexpression reduces intracellular ROS levels, thereby inhibiting ferroptosis in diffuse large B-cell lymphoma [66]. The NRF2 and NRF1 pathways regulate GPX4 expression, with NRF2 downregulation elevating GPX4 levels and NRF1 enhancing GPX4 expression to inhibit ferroptosis. Forcina et al. showed that NRF1 and NRF2 can compensate for each other’s deficiencies, highlighting the complex interplay between these pathways and ferroptosis [67]. The cellular lipid antioxidant defense system plays a dual role in modulating ferroptosis in GBM. ACSL4, a key enzyme in lipid peroxidation, promotes ferroptosis by preferentially incorporating polyunsaturated fatty acids into membrane phospholipids [68]. Its activity is modulated by HSP27 through post-translational stabilization [69]. Li et al. have demonstrated that the NF-κB pathway cooperates with GPX4 inhibition to induce ferroptosis, as GPX4 suppression alone proves insufficient in GBM cells [70, 71]. NF-κB activation also contributes to the mesenchymal (MES) transition phenotype associated with treatment resistance and tumor recurrence [72].

These interconnected pathways—involving iron metabolism (TRIM7, COPZ1, IRP-IRE), antioxidant defenses (NF-κB, Nrf2, APOC1), and lipid peroxidation regulation (ACSL4, PRRX2/GCH1/BH4)—collectively determine ferroptosis susceptibility in GBM. Therapeutic strategies targeting these mechanisms may provide novel approaches for GBM treatment by selectively inducing ferroptosis in tumor cells while sparing normal tissues. The complexity of these regulatory networks underscores the need for combination therapies that simultaneously target multiple nodes in the ferroptosis pathway for optimal therapeutic efficacy against this aggressive brain tumor. Table 1 summarizes the mechanisms of these targets and their relationship with GBM and ferroptosis.

**Table 1:** Mechanisms of each target and their relationships with ferroptosis.

Target	Mechanism	Relationship	Effect	Ref.
TRIM7	-Ubiquitinates NCOA4 -Inhibits ferritinophagy -Deduces ferritin degradation -Decreases intracellular free iron	-Iron metabolism regulation	-Intracellular iron↓	[61]
COPZ1	-Deduces ferritin degradation	-Iron metabolism regulation	-Intracellular iron↓	[31, 62]
IRP/IRE Signaling Pathway	-Inhibit the translation of ferritin and FPN -Reduces ferritin storage and iron efflux	-Iron metabolism regulation	-Intracellular iron↑	[64]
ACSL4	-Promotes the conversion of unsaturated fatty acids and lipid peroxidation	-Antioxidant defense system	-AA-CoA↑ADA-CoA↑ -Lipid peroxidation↑	[111]
Nrf2-ARE Pathway	-Antioxidant -Activates the expression of a range of cytoprotective genes	-Antioxidant defense system	-ROS↓	[112]
NF-κB pathway	-Assists RSL3 in inhibiting GPX4 activity -Regulates the expression of iron metabolism-related proteins -Modulates antioxidant responses or stress signaling pathways -Regulates MES transition and invasion	-Lipid metabolism and peroxidation regulation	-Intracellular iron↑ -GPX4↓ -MES transformation↑	[70]
APOC1	-Upregulates HO-1 and NQO1 -Increase GSH and GPX4 expression	-Lipid metabolism and peroxidation regulation	-CBS↑ -GSH↑	[52]
PRRX2/GCH1/BH4	-Increases lipid peroxidation and BH4	-Lipid metabolism and peroxidation regulation	-BH4↑	[54, 113, 114]

## Ferroptosis-based treatment for GBM

GBM, the most aggressive primary brain tumor in adults, is treated with surgery, radiation, and chemotherapy. Given ferroptosis’s link to GBM, novel therapies like targeted therapy and nanomedicine have emerged. Here, we focus on ferroptosis-based targeted and nanomedicine treatments for GBM.

### Targeted therapy

The ferroptosis pathway can be broadly divided into GPX4-dependent and GPX4-independent pathways. Therefore, targeting these pathways is currently the most commonly used therapeutic strategy. The main pathways of ferroptosis in GBM include iron metabolism, the GPX4 pathway, the FSP1 pathway, and lipid metabolism. We will introduce the corresponding targeted therapy strategies from these four aspects.

Iron plays a crucial role in ferroptosis, primarily by catalyzing the generation of ROS. Drugs such as DFOM and deferiprone can chelate iron and thereby inhibit ferroptosis. In the context of GBM, reducing iron levels may potentially decrease ferroptosis, rendering GBM cells more resistant to therapies that induce this form of cell death. Modulating ferritin, the primary intracellular iron storage protein, may help balance iron levels within GBM cells and influence their susceptibility to ferroptosis.

GPX4 is a pivotal regulator of ferroptosis, functioning as a selenoprotein that directly reduces cytotoxic phospholipid hydroperoxides (PLOOH) to benign phospholipid alcohols, thereby preventing lethal lipid peroxidation. Consequently, targeted inhibition of GPX4 represents a core therapeutic strategy for inducing ferroptosis in GBM cells. Small-molecule inhibitors like RSL3 and FIN56 directly bind to and inhibit GPX4 enzymatic activity. This inhibition leads to the accumulation of PLOOH, a surge in ROS, and ultimately drives GBM cells into ferroptosis. Beyond direct inhibition, compounds can exert complex, context-dependent effects on the GPX4 pathway. Dihydroartemisinin (DHA) exemplifies this duality: at low concentrations or under specific conditions, DHA can paradoxically increase GPX4 activity by upregulating ATF4 expression, potentially suppressing ferroptosis. Conversely, at higher concentrations or in specific cellular contexts, DHA induces endoplasmic reticulum stress in glioma cells. This stress activates the PEAK-HSPA5-GPX4 signaling axis, involving upregulation of PERK, activation of ATF4, and subsequent induction of HSPA5. While HSPA5 upregulation can enhance GPX4 activity and inhibit lipid peroxidation, experimental disruption of this pathway (e.g., via siRNA) blocks DHA’s protective effect, allowing its inherent ROS-generating capacity to prevail, ultimately inducing ferroptosis [73]. In stark contrast, RSL3 functions primarily as a direct and potent GPX4 inhibitor. Its mechanism consistently involves suppressing GPX4 activity, elevating ROS levels, and downregulating ATF4 expression [74]. Furthermore, RSL3 activates the intracellular NF-κB pathway, which contributes to further ATF4 downregulation, amplifying the ferroptotic cascade [9]. The critical role of GPX4 extends beyond direct ferroptosis induction; inhibiting GPX4 is also a promising strategy to overcome tumor cell resistance to conventional chemotherapy drugs by triggering ferroptosis as an alternative cell death mechanism [75]. Given GPX4’s central position within the primary antioxidant defense against ferroptosis, mediated through the cystine/glutamate antiporter system (System Xc-) and GSH synthesis, it remains the predominant focus for targeted ferroptosis-inducing drugs. Enhancing the efficacy and specificity of these GPX4-targeting agents, particularly within the challenging brain TME, is an active area of research. Innovative drug delivery systems are being developed to improve the bioavailability and tumor targeting of GPX4 inhibitors like RSL3 and FIN56, while minimizing off-target effects on normal tissues. This targeted approach, selecting drugs to precisely disrupt key nodes like GPX4 within the ferroptosis pathway, is a cornerstone of current therapeutic strategies against GBM.

FSP1 functions as an alternative pathway to GPX4, protecting cells from ferroptosis. Novel small molecules targeting FSP1 are currently under investigation to induce ferroptosis in GBM cells. However, enhancing FSP1 activity may be detrimental to GBM treatment strategies, as FSP1 counteracts ferroptosis. Recent studies have shown that FSP1 effectively prevents ferroptosis caused by GPX4 deletion or inhibition, and its expression increases when GPX4 expression is reduced. As a ferroptosis resistance factor, FSP1 compensates for GPX4 deletion by mediating the reduction of oxidized coenzyme Q to ubiquinol, a lipophilic antioxidant, thereby preventing the propagation of lipid peroxidation [76, 77]. Since FSP1 expression is associated with resistance to GPX4 inhibitors, FSEN1, a potent FSP1 inhibitor, enhances the sensitivity of cancer cells to ferroptosis inducers targeting the GSH-GPX4 axis [78]. Therefore, FSP1 inhibitors can be combined with GPX4-targeting agents such as DHA for the treatment of GBM.

Lipid metabolism is closely linked to ferroptosis, as it involves the generation and accumulation of lipid peroxides. Drugs that enhance lipid peroxidation, such as artemisinin derivatives, are being explored for their potential to induce ferroptosis in GBM cells. Orlistat and other fatty acid synthase inhibitors can disrupt lipid metabolism, promoting the accumulation of toxic lipids and thereby triggering ferroptosis.

Combining ferroptosis inducers with existing GBM therapies may enhance treatment efficacy. Some drugs can also act on multiple pathways to induce ferroptosis. Lipid peroxidation is caused by oxidative attacks on the carbon–carbon double bonds in lipids. ACSL4 and LPCAT3 are key factors driving ferroptosis through lipid peroxidation [68, 79]. ACSL4 enhances the sensitivity of tumor cells to ferroptosis by modulating lipid composition [68], thereby inhibiting cell proliferation through ferroptosis [80]. Dihydrotanshinone I (DHI) [81], capsaicin [82], and boric acid (BA) [83] simultaneously target GPX4 and ACSL4, reduce GSH and GPX4 levels, and induce ferroptosis in tumor cells. Li Mei et al. demonstrated through comparative experiments that DHI significantly increases lipid peroxidation levels and inhibits GSH, leading to a decrease in the GSH/GSSG ratio and weakened cellular antioxidant capacity, further resulting in the loss of GPX4 activity. Therefore, DHI can downregulate GPX4 and upregulate ACSL4 [81]. Capsaicin increases ACSL4 mRNA and protein levels in U87-MG and U251 cells while reducing GPX4 mRNA and protein levels, thereby decreasing intracellular GPX4 and GSH levels, increasing ACSL4 levels, and exhibiting concentration-dependent anti-proliferative effects [82]. These findings indicate that different drugs can induce ferroptosis in tumor cells by acting on distinct targets and modulating the expression of various receptors (Table 2).

**Table 2:** Mechanisms and signaling pathways of targeted drugs treating GBM through ferroptosis.

Drug	Target	Pathway	Effect	Ref.
DHA	-GPX4	-PERK/ATF4/HSPA5 pathway	-GPX4↑ -ATF4↑	[115]
RSL3	-GPX4	-NF-κB pathway	-GPX4↓ -ATF4↓ -ROS↑	[74]
Dihydrotanshinone I	-GPX4 -ACSL4	-GPX4 and ACSL4 pathway	-GSH/GSSG↓ - GPX4↓ -ACSL4↑	[81]
Capsaicin	-ACSL4 -GPX4	-GPX4 and ACSL4 pathway	-GPX4↓ -ACSL4↑ -GSH↓	[82]
Boric acid (BA)	-ACSL4 -GPX4	-ACSL4/GPx4SEMA3F/NP2 pathways	-GPX4↓ -ACSL4↑	[83]
Roxadustat (RXD)	-HIF-α	-lipid peroxidation pathway	-GPX4↓ -4-HNE↑	[116]
FSEN1	-FSP1	-GSH-GPX4 axis	-GPX4↓	[78]

### Immunity therapy

Cell death can be categorized into accidental cell death and RCD. As a non-apoptotic RCD, ferroptosis is involved in the survival, differentiation, activation, and migration of immune cells, and it influences tumor growth by modulating immune responses [84, 85].

Immunotherapy kills tumor cells through the human immune system. Due to its minimal adverse effects compared to other chemotherapeutic drugs, it is currently a widely studied novel treatment approach [86]. Ferroptosis in tumor cells triggers damage-associated molecular patterns (DAMPs), releasing cytokines to stimulate adaptive immunity, thereby promoting the immunogenic cell death (ICD) of tumor cells [87]. During ICD, DAMPs and pathogen-associated molecular patterns bind to pattern recognition receptors, generating anti-tumor immune responses. On the other hand, ferroptosis promotes the release of cytokines and chemokines, triggering inflammatory responses and facilitating tumor growth.

Tregs, myeloid-derived suppressor cells, M1/M2 macrophages, and other immunosuppressive cells within the TME can resist ferroptosis by highly expressing GPX4, thereby maintaining the activation and survival of tumor cells. GPX4 inhibits lipid peroxidation by reducing lipid hydroperoxides to non-toxic lipid alcohols, thereby preventing ferroptosis. Treatment of these cells with lapatinib, statins, trigonelline, or through genetic editing to suppress GPX4 expression can induce ferroptosis and reverse their tumor-promoting functions [28, 58, 88]. Notably, indoleamine 2,3-dioxygenase 1 (IDO1), a pivotal enzyme in tryptophan catabolism, drives immunosuppression by converting tryptophan to kynurenine (Kyn), thereby activating the aryl hydrocarbon receptor (AhR). In GBM, elevated IDO1 expression correlates with tumor progression and poor prognosis, partly by suppressing ferroptosis via stabilizing SLC7A11 mRNA through FTO-dependent m6A methylation [89]. The IDO1-Kyn-AhR axis facilitates AhR nuclear translocation, which transcriptionally represses FTO, leading to m6A hypermethylation and stabilization of SLC7A11 transcripts. Clinically, IDO1 inhibitors, evaluated in colorectal, pancreatic, and bladder cancers, demonstrate the potential to restore ferroptosis sensitivity and mitigate radiotherapy-induced immunosuppression in GBM. Combinatorial strategies targeting IDO1 with m6A modulators may synergistically enhance therapeutic efficacy by concurrently inducing ferroptosis and reversing immune evasion [90].

Additionally, these therapeutic approaches can promote immune cells to release cytokines that enhance the ferroptosis activity of tumor cells. For instance, interferon-γ (IFN-γ) released by cytotoxic T cells (CTLs) activates the downstream JAK-STAT1 signaling pathway, downregulating the expression of the cystine/glutamate antiporter system (xc system), thereby reducing intracellular GSH synthesis, increasing free iron levels, and ultimately inducing ferroptosis in GBM cells [91]. To prevent anti-tumor immunity from transitioning into an immunosuppressive response when the number of ferroptotic cells reaches a certain threshold in the TME, it is necessary to inhibit the activity of immune cells such as natural killer (NK) cells and T cells. Prostaglandin E2 (PGE2), as a key immunosuppressive mediator, can suppress the activity of CTLs [92], dendritic cells [91], and NK cells [93], thereby interfering with anti-cancer immune responses. Studies have shown that T cell function is significantly inhibited under high ROS conditions, while their cytotoxicity is restored under low ROS conditions [94]. Metformin, as a ROS inhibitor, can maintain T cell activity by reducing intracellular ROS levels, thereby promoting tumor clearance and reducing tumor-associated inflammation [95]. Furthermore, metformin inhibits the differentiation of naive CD4+ T cells into Tregs, reducing Treg infiltration in tumor tissues and ensuring the normal function of anti-tumor immune mechanisms [96].

In addition, the presence of M2 macrophages is closely associated with increased invasiveness of GBM and plays a critical role in the acquisition of chemotherapy and radiation resistance in GBM cells [28, 88]. M2 macrophages support tumor growth and metastasis by secreting immunosuppressive cytokines, such as IL-10 and TGF-β, and promoting angiogenesis and stromal remodeling. Solute carrier family one member 5(SLC1A5), functioning as a glutamine transporter on the cell membrane [88], transports the key metabolite α-ketoglutarate during the tricarboxylic acid cycle, thereby increasing intracellular oxidative stress levels. SLC1A5 is highly expressed in GBM and various other cancers as a ferroptosis-inducing gene [97]. Studies have shown that SLC1A5 is closely associated with the activation of immune cells, particularly macrophages, and can inhibit anti-tumor immune responses by regulating the osmotic state and metabolic activity of TAMs in the TME.

Furthermore, CD4+ and CD8+ T cells in the TME often exhibit functional impairment, inactivation, or exhaustion, and frequently co-express multiple immune checkpoint molecules, such as PD-1, lymphocyte activation gene 3, and T cell immunoglobulin and mucin domain-containing protein 3 (TIM-3). These immune checkpoint molecules promote tumor immune escape by inhibiting T-cell activation and function. CD8+ T cells can downregulate the expression of SLC7A11, inhibit the glutamate-cysteine cycle, and reduce cysteine uptake in tumor cells, thereby promoting lipid peroxidation and ferroptosis [7]. CD8+ T cells release IFN-γ through immune responses, which synergize with radiation therapy-activated TAMs to further suppress SLC7A11 expression and promote tumor lipid oxidation-induced ferroptosis [93]. CD36, a fatty acid transporter, increases the uptake of fatty acids by CD8+ T cells in the TME, leading to the inhibition of their effector function, reduced production of cytotoxic factors, such as perforin and granzymes, and weakened anti-tumor capacity. Therefore, targeting CD36 can restore T cell-dominated immune mechanisms in vivo and enhance anti-tumor effects by promoting ferroptosis in tumor cells [98].

Due to the rapid proliferation of GBM, the TME is suppressed by factors such as acidity and hypoxia, rendering immunotherapy ineffective [95, 96]. It can be inferred that utilizing ferroptosis to treat tumors requires enhancing the anti-tumor efficacy of drugs, and currently known agents capable of achieving this goal include calcium ions. Calcium ions can be transported into tumor cells through the ferroptosis pathway and induce immune responses within tumor cells. However, it is important to note that ferroptosis is a double-edged sword. Under drug induction, appropriate ferroptosis can inhibit tumor growth; however, excessive ferroptosis leads to cellular hypoxia and nutrient deprivation, triggering inflammation-related immunosuppression within the TME and thereby promoting tumor cell proliferation. Thus, controlling ferroptosis, preventing excessive cell death, and developing drugs that selectively kill tumor cells without disrupting the immune microenvironment are key challenges requiring further research.

### Nanomedicine therapy

The BBB, comprising endothelial cells, pericytes, and astrocytes, is essential for maintaining CNS homeostasis but poses a formidable obstacle to GBM therapy [99]. Its selective permeability, governed by tight junctions, transport proteins, and metabolic enzymes, severely restricts brain penetration of macromolecular therapeutics, necessitating high systemic doses that cause hepatotoxicity and immunosuppression. [100, 101]. Nanomedicine offers transformative solutions to this challenge through engineered delivery systems. Nanoparticles (NPs) exploit both passive diffusion and active receptor-mediated transcytosis to traverse BBB gaps (<1 nm), achieved by optimizing size, surface charge, and hydrophobicity while incorporating targeting ligands (e.g., antibodies, transferrin) that bind endothelial receptors [100]. This enables tumor-selective accumulation of ferroptosis-inducing payloads while minimizing off-target effects.

Beyond BBB penetration, nanoplatforms facilitate multi-level targeting of ferroptosis pathways in GBM. Nanoencapsulation allows the co-delivery of synergistic agents, such as GPX4 inhibitors (RSL3, FIN56), iron donors, and lipid peroxidation amplifiers, at reduced individual doses to circumvent toxicity thresholds and overcome resistance mechanisms [102, 103]. Hydrogel-based systems further enhance spatial control; when implanted in tumor resection cavities, these biocompatible matrices provide sustained, localized release of ferroptosis inducers, bypassing systemic exposure [104]. Biomimetic strategies exemplify this precision: macrophage membrane-coated nanogels leverage innate BBB-transmigrating capabilities while evading immune clearance, prolonging circulation time, and enhancing tumor adhesion without compromising drug-loading capacity [105, 106]. Gold, mesoporous silica, and upconversion NPs serve as ideal cores for such platforms due to their stability and multifunctionality [7].

Current nanomedicine approaches for ferroptosis induction converge on two strategic paradigms. First, nanocarriers directly deliver ferroptosis modulators to molecular targets, depleting GSH via cystine disruption, inhibiting GPX4, or elevating labile iron pools to amplify lipid peroxidation. For instance, the AZA-BD@PC NP platform depletes intracellular cysteine to disrupt GSH synthesis while generating H₂S. This dual action creates an acidic microenvironment conducive to ferroptosis, dysregulates glycolysis, and triggers iron accumulation, simultaneously activating p53-mediated apoptosis and DNA damage pathways for potent anti-GBM effects [107]. Second, nanozymes exploit catalytic activities to induce ferroptosis biocatalytically. Nanase (Cox-MION), a peroxidase-mimetic nanoconjugate, directly generates lipid peroxides through enzyme-like activity while its spherical structure mechanically suppresses tumor growth, achieving ~40% volume reduction in preclinical models [108].

Complementary strategies include convection-enhanced delivery (CED) systems that enhance parenchymal drug distribution and chemical modifications (e.g., lipid group conjugation) to improve drug lipophilicity for BBB transit [109, 110]. Collectively, these innovations position nanomedicine as a versatile framework for overcoming biological barriers, enabling precise ferroptosis manipulation, and advancing combination therapies against GBM’s heterogeneity and resistance. Future developments will likely integrate real-time imaging, stimuli-responsive drug release, and personalized biomimetic designs to fully exploit ferroptosis vulnerabilities in clinical translation.

## Conclusions and perspectives

GBM, a highly aggressive CNS tumor, remains a major therapeutic challenge due to its resistance to conventional treatments. Ferroptosis, an iron-dependent cell death mechanism distinct from apoptosis, offers a promising therapeutic avenue. Key regulators like GPX4 and system Xc^-^ control ferroptosis, and their inhibition (e.g., via RSL3 or erastin) enhances GBM cell susceptibility. Chemotherapy and radiation can also induce ferroptosis by disrupting redox balance. Nanotechnology enables targeted delivery of ferroptosis inducers, improving efficacy and reducing toxicity. Collectively, these advancements underscore the potential of ferroptosis as a therapeutic target and highlight the importance of integrating innovative technologies to improve outcomes in GBM treatmentHowever, clinical translation faces hurdles, including GBM heterogeneity, the TME, and lack of reliable biomarkers. Resistance mechanisms, such as GPX4 upregulation, further complicate treatment. Future strategies should combine ferroptosis inducers with immunotherapy, optimize iron-modulating agents, and refine nanocarrier delivery systems. Multidisciplinary efforts are essential to advance ferroptosis-based therapies and personalize GBM treatment, ultimately improving patient outcomes. Looking ahead, the development of innovative and multifaceted strategies is imperative to address the complex challenges associated with GBM management and to fully harness the therapeutic potential of ferroptosis. A particularly promising approach involves the rational design of combination therapies that synergistically integrate ferroptosis inducers with emerging immunotherapeutic modalities, such as immune checkpoint inhibitors, chimeric antigen receptor T-cell therapies, or cancer vaccines. This combinatorial strategy may not only enhance anti-tumor immune responses through the release of DAMPs and tumor-associated antigens from ferroptotic cells but also simultaneously induce selective and potent cell death in GBM, potentially overcoming therapeutic resistance mechanisms. Further investigation is warranted to identify and characterize novel small-molecule compounds or targeted agents that can selectively induce ferroptosis in GBM cells while sparing normal neural tissue. High-throughput screening platforms, computational drug discovery approaches, and structure-activity relationship studies should be employed to optimize the specificity and potency of these agents. Additionally, the development of pharmacological agents that precisely modulate intracellular iron homeostasis, such as iron chelators or iron oxide NPs, or enhance iron bioavailability specifically within the TME, may facilitate the targeted exploitation of ferroptosis pathways. These agents should be designed to exploit the unique metabolic vulnerabilities of GBM cells, including their altered iron metabolism and redox homeostasis. Furthermore, the optimization of ferroptosis inducer delivery through advanced nanomedicine approaches, such as engineered nanocarriers, tumor-targeted exosomes, or BBB-penetrating delivery systems, could significantly enhance therapeutic efficacy while minimizing systemic toxicity and off-target effects. These delivery platforms should incorporate stimuli-responsive release mechanisms, such as pH-sensitive or redox-sensitive components, to ensure precise spatiotemporal control of drug release within the TME. Additionally, the integration of real-time imaging and monitoring capabilities into these delivery systems could enable personalized treatment adjustments and improve therapeutic outcomes. Therefore, sustained investment in basic and translational research, coupled with robust interdisciplinary collaboration, will be pivotal in transforming ferroptosis modulation from a promising experimental concept into a clinically viable therapeutic paradigm for GBM.

## Data Availability

Data will be made available on request.
